# Narrative Review of COVID-19 Impact on Obsessive-Compulsive Disorder in Child, Adolescent and Adult Clinical Populations

**DOI:** 10.3389/fpsyt.2021.673161

**Published:** 2021-05-13

**Authors:** Vittoria Zaccari, Maria Chiara D'Arienzo, Tecla Caiazzo, Antonella Magno, Graziella Amico, Francesco Mancini

**Affiliations:** ^1^School of Cognitive Psychotherapy, Rome, Italy; ^2^Department of Human Sciences, Marconi University, Rome, Italy

**Keywords:** COVID-19, coronavirus, obsessive-compulsive disorder, obsessive-compulsive symptoms, adults, children, adolescents, narrative review

## Abstract

**Background:** The COVID-19 pandemic and quarantine had a significant impact on mental health which resulted in an increase of anxiety and depression in adult, child and adolescent clinical populations. Less is known about the potential effect of pandemic on obsessive-compulsive disorder (OCD) so there is a lack of review work to illustrate the impact of the COVID-19 pandemic on OCD.

**Purpose:** The main objective is to review all the empirical contributions published after March 2020 that dealt with the impact of the COVID-19 pandemic on OCD in adults, children and adolescents, investigating the state-of-the-art literature concerning the impact on OCD and detailing limitations.

**Methods:** The literature search was conducted using PsycINFO, PsycARTICLES, MEDLINE, Scopus, Web of Science, PubMed, and Google Scholar. This review analyzed all studies from January 2020 to 8 January 2021, focusing on clinical populations of children, adolescents, and adults with OCD.

**Results:** A total of 102 articles were screened, resulting in the identification of 64 full-text articles to be further scrutinized. Upon closer examination, there was consensus that 39 articles met the study inclusion criteria and 14 of these were selected for study. Analysis of the results revealed that COVID-19 had an impact on OCD in both adults and young people and seems to have caused exacerbation of symptoms, especially of the contamination/washing subtypes. Eight studies in adult samples showed an increase in the severity of obsessive-compulsive symptoms; two studies underlined a minimal impact of COVID-19 on OCD patients and one study showed a slight improvement in symptoms. Two out of three studies on children and adolescents showed an exacerbation of OCD and a worsening even in the presence of an ongoing treatment.

**Conclusions:** The studies reviewed are few. There are more studies on adult OCD than on children and adolescents. The results are controversial: few studies examined OCD subtypes; in most studies the typology of treatment was not clear and the samples covered a wide age range; a large number of studies did not use the same monitoring period or quantitative measures, both of which make it difficult to compare or rely on the results.

## Introduction

The COVID-19 pandemic and consequent quarantine had a significant impact not only on physical health but also on mental health in both the clinical and the general population. Indeed, there is a wide consensus that the COVID-19 pandemic has led to worldwide measures with severe consequences for millions of people (1–3). Several studies show, in fact, how this event generated a degree of malaise and psychological distress in the general population (4–12), in the adult clinical population (13, 14) and in children and adolescents (15–19), showing a worsening of various clinical pictures and an increase in psychological difficulties.

Various psychological problems and important consequences in terms of mental health emerged progressively, including anxiety, stress, depression, suicidal risk, frustration and uncertainty during the outbreak, (20–26). Moreover, the COVID-19 pandemic has produced an increase in psychiatric disorders (e.g., depressive and anxiety disorders, post-traumatic stress disorder) as well as grief-related symptoms such as complicated grief disorder (1, 19, 27, 28).

Although the consequences of the COVID-19 pandemic for general mental health and the increase in anxiety and depression are clear, less is known about the potential effect of the pandemic on obsessive-compulsive disorder (OCD). OCD is a common, chronic and long-lasting disorder in which a person has uncontrollable, reoccurring thoughts (obsessions) and/or behaviors (compulsions) that he/she feels the urge to repeat over and over and it is one of the most disabling psychiatric disorders, with a prevalence of around 2% (29). The increase in distress, concern and fear has affected reactions to present situations and exacerbated some existing psychiatric issues because some symptomatic domains have been triggered, typically OCD (30, 31).

In this situation, the health impact of the COVID-19 pandemic on OCD cannot be overlooked. A growing body of research has shown, in fact, how OCD is associated in some cases with a symptomatology that is highly sensitive to the fear and probability of contamination, with the perception of a greater possibility of becoming infected or infecting others and with protective behaviors aimed at removing or neutralizing the possible risk of contamination (32–39), driven by the goal of preventing or neutralizing guilt for irresponsibility, a specific mental state related to checking and cleaning compulsions (40–42). All these aspects were strongly conveyed in this period of emergency due to COVID-19.

In relation to OCD, a few studies have been published to date that highlight how some obsessive-compulsive (OC) symptoms have worsened due to the current situation in both adult (43–46) and young clinical populations (47, 48). The precautionary measures against COVID-19, such as hand washing, maintaining a high level of hygiene and avoiding handshakes, may have triggered psychological distress in OCD patients, consequently increasing their symptoms.

However, at present, there is a lack of review work to illustrate the impact of the COVID-19 pandemic on OCD patients or to highlight in which profiles clinical worsening has occurred, which symptom areas have suffered exacerbation and in what period they were detected. The consequences of the pandemic on OCD in adults, children and adolescents are not clear and it is therefore essential to verify and analyze the extent of the impact on OCD in terms of worsening of symptoms and to verify which symptoms, variables or cognitive ingredients are involved.

### Research Question

The purpose of the present narrative review is to investigate state-of-the-art literature concerning the impact of the COVID-19 pandemic on OCD patients and to highlight their limitations. In particular, we want to verify if there has been a worsening of OC symptoms and which subtypes of OCD are most involved. The main objective is to analyze all the empirical contributions published after March 2020 that dealt with the impact of the COVID-19 pandemic on OCD in adults, children and adolescents and to provide a synthesis of the current literature. We discuss findings from studies that analyze the impact of COVID-19 in OCD according to the most recent published reviews [e.g., (49, 50)] that provide insight into the pandemic's implications for OCD symptoms up until last summer. For instance, in their review Sulaimani and Bagadood (49) assessed various sources regarding OC symptoms and the pandemic via a study of literature related to OCD conditions. Their results showed that anxiety and the associated prevention measures increased the severity of OCD symptoms. Other precautionary measures against COVID-19, such as constant hand washing, maintaining a high level of hygiene, avoiding handshakes and not touching the face, trigger psychological distress in OCD patients and consequently increase their symptoms. However, this study refers only to USA, China, India and UK so it is not possible to generalize these results.

It appears important for clinicians and the scientific community to shed light on the impact of this event on OCD, a psychiatric disorder that causes significant impairment in general functioning. This knowledge is fundamental to make use of more appropriate and timely interventions in clinical practice and understand how contextual variables can exacerbate some OC symptoms. We argue that research on OCD in times of pandemics is necessary because such global situations could be prolonged or repeated.

## Method

This review analyzed all studies from January 2020 to 8 January 2021 concerning OCD and the coronavirus pandemic, focusing on clinical populations of children, adolescents and adults with OCD. The aim was to review existing contributions illustrating the coronavirus pandemic's impact on OCD symptoms. We included all studies that investigated the impact of the COVID-19 pandemic on OCD in children, adolescents and adults. We reached information from studies focusing on different countries. After an initial screening, we included data from a large range of countries, such as: India, Germany, Japan, Iran, Ireland, Netherlands, Turkey, Denmark, and Israel. This provides a wide view on distinct political, cultural, economic variables concerning the impact of COVID-19 pandemic on OC symptoms.

The literature search was conducted using the following databases: PsycINFO, PsycARTICLES, MEDLINE, Scopus, Web of Science, PubMed and Google Scholar. Keywords searched in order to find our results were: “OCD,” “coronavirus,” “pandemic,” “COVID-19,” “sars-cov-2,” “OCD symptoms,” “obsessive-compulsive disorder,” “adults,” “children,” and “adolescents,” used in different combinations.

### Eligibility Criteria

The selection of studies in the narrative review was decided according to the following inclusion criteria: peer-reviewed academic journals published between January 2020 and 8 January 2021; empirical study on clinical OCD sample and impact of the COVID-19 pandemic in a population of children and/or adolescents and/or adults; cross sectional or longitudinal study design; and articles with accessible abstracts and full text. Exclusion criteria were: not providing original contributions (e.g., review, comment, or letter to the editor); providing exclusively qualitative data; and studies conducted on the general population. Typology of treatment, presence of comorbidity, published status and language of the contribution were not exclusion criteria, and nor were gender composition, ethnicity and nationality of the sample.

### Search Strategy

**Figure 1:**
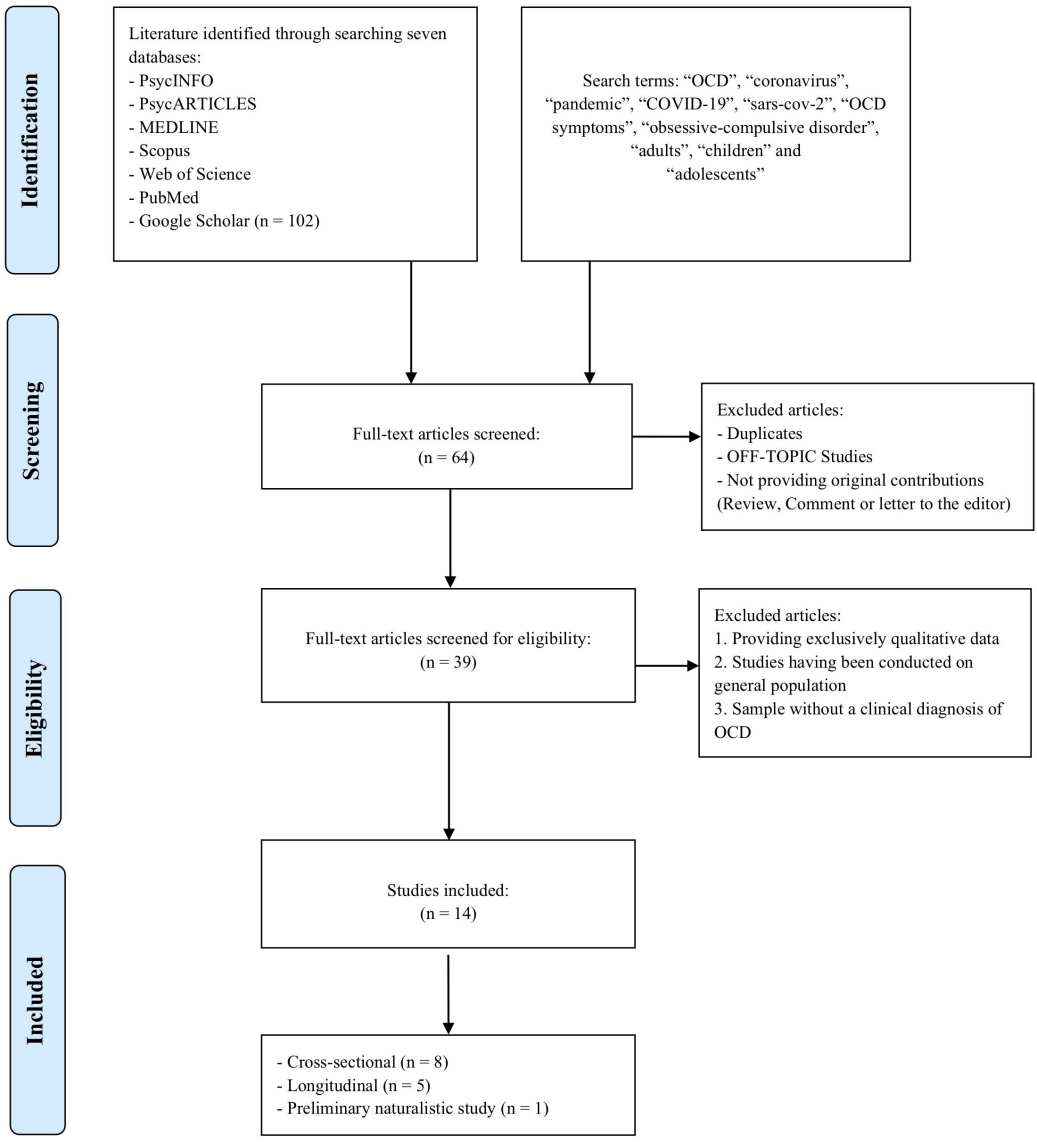
Flowchart on search strategy.

Articles were read and assessed for relevance. In total, 102 articles on COVID-19 and OCD were reviewed; however, 88 articles were excluded because they were literature reviews, essays or did not represent the target population. Thus, we selected 14 articles that met all the inclusion criteria (Figure 1). The characteristics of the reviewed articles are summarized in Tables 1, 2. Data and measures not relating to OC symptoms were omitted from the tables.

**Table 1:** Studies among sample of adults.

**References/Country**	**Aims/Purpose**	**Research design**	**Sample characteristics**	**% Males**	**Comorbidity**	**Treatment**	**Outcome/Measures**	**Monitoring period**	**Main findings**
Chakraborty and Karmakar (44), India	Assess the impact of COVID-19 on patients who already have OCD, particularly obsession of contamination and washing compulsion	Longitudinal study	*N* = 84, Age: *NS*	23.8	*NS*	Regular pharmacological treatment 57 Irregular pharmacological treatment 13	Phone interview, Y-BOCS	April–May 2020	No increase in obsessive and compulsive symptoms. 6% reported symptoms exacerbation (they were not taking their medications)
Storch et al. (51), Texas (USA)	Evaluate clinicians' perspectives regarding the impact of the COVID-19 pandemic on individuals with OCD receiving ERP under their care prior to and during the pandemic	Longitudinal study	Clinician (respondent) information: *N* = 137, Age: 23–73 Reported patient information: *N* = 232, Age: 4–77^*^	21.9 46.5	Anxiety disorder 110 (48%) Depressive disorder 75 (32%)	ERP 232	Online survey Questionnaire adapted from: NIMH-GOCS and Y-BOCS	July–August 2020	Clinicians estimated that 38% of their patients had symptoms worsening. Individuals with negative financial impacts from the COVID-19 had increases in OC symptoms
Jelinek et al. (52), Germany	Assess the influence of the COVID-19 pandemic on persons with OCD, in particular people with washing compulsions (“washers”) in regard to change in symptom severity, the reasons for the change, and dysfunctional as well as functional beliefs during the COVID-19 pandemic	Longitudinal study	*N* = 394, Age: 37.76 (12.14) Subsamples: *N* = 223, washers, Age: 37.43 (11.52) *N* = 171, not-washers, Age: 38.20 (12.92)	25.6 21.1 31.6	*NS*	*NS*	Online survey Qualitative questionnaire OCI-R	March–May 2020	Increase in the severity of OCD and in the number of obsessions: especially for washers in comparison to not-washers. Washers agreed more than not- washers with the hygiene related dysfunctional beliefs. Hygiene-related dysfunctional beliefs were associated with an increase in OC symptoms severity
Prestia et al. (53), Italy	Evaluate the changes on OCD symptoms in a group of patients with OCD. Assess the effects of contamination symptoms and remission state before the quarantine on OCD symptoms change during the quarantine, controlling for some variables related to the life in quarantine	Preliminary naturalistic study	*N* = 30, Age: 20–73 (14.87)	46.6	Mood disorder 2 Personality disorders 3 Any psychiatric comorbidities before the quarantine 5	Pharmacological treatment 30	Y-BOCS-SC Qualitative questionnaire	January–April 2020	13.3% of the twelve patients in complete remission on OC symptoms, returned to clinically significant OCD. Increase in the severity of total OC symptoms. Elevated OC symptom worsening in people with contamination symptoms and living with a relative
Capuzzi et al. (54), Italy	Assess clinical characteristics of patients receiving psychiatric consultations during the lockdown in two psychiatric emergency services and to compare them to the same period	Cross sectional study	Period A (2019): *N* = 388 (2 with OCD), Age: 43.9 (16.5) Period B (2020): *N* = 225 (9 with OCD), Age: 44.2 (18.1)	49.2 51.5	*NS*	Pharmacological treatment 248 Pharmacological treatment 151	Clinical data	Period A (2019) February–May Period B (2020) February–May	Decrease in the number of psychiatric emergency consultations during the lockdown period. Higher psychiatric emergency visits during the lockdown in OCD patients
Matsunaga et al. (55), Japan	Investigate the impact of the COVID-19 pandemic on the changes of OCD severity or symptomatology	Cross sectional study	*N* = 24 fully remitted *N* = 36 partially remitted Age: >18 (41.5)	25	*NS*	*NS*	Y-BOCS	April–May 2020	10% experienced the deterioration of the OC symptom severity. No significant differences between the fully remitted 8.3% and the partial remitted 11.1% groups. No subjects exhibited the symptom transition of their principal symptoms
Benatti et al. (56), Italy	Describe the impact of COVID-19 pandemics within a sample of Italian patients affected by OCD	Cross sectional study	*N* = 123, Age: 16–65^*^	54	*NS*	Pharmacological treatment 123	Phone interview Psychiatric interview	March–May 2020	More than 1/3 of sample showing a clinical worsening of OCD and reported a significant emergence of new obsessions and compulsions with an exacerbation of past one
Khosravani et al. (57), Iran	Validate the Persian-COVID Stress Scale in Iranian patients with anxiety disorders and OCD and to compare COVID-19 related stress responses	Cross sectional study	*N* = 300, Age: 17–67 (11.86)	40.3	Major depressive disorders 72 Anxiety disorder 54	*NS*	VOCI OCI-R OCS	June–August 2020	OCD patients had higher COVID-19 related stress responses, such as: fear of danger and contamination, socio-economic consequences, xenophobia, traumatic stress and compulsive behaviors of checking and reassurance-seeking
Kuckertz et al. (58), United States	Challenge the notion that by definition OCD patients will fare worse than the general public or that ERP cannot proceed effectively during this time	Longitudinal study	*N* = 8, Age: *NS*	*NS*	*NS*	ERP 8 ACT 8 CBT 8 Psychiatric treatment 8	Y-BOCS PSWQ-A DOCS	January–May 2020	37% considered COVID-19 as an interesting opportunity to be more fully engaged in exposure. 12.5% exacerbation of OC symptoms. 37% required modifications to their treatment plan due to increased restrictions
Plunkett et al. (59), Ireland	Examine the psychological impact of the COVID-19 pandemic on patients with established anxiety disorders	Cross sectional study	*N* = 30 (12 with OCD), Age: 38.8 (12.8)	40	Personality disorder 5 Schizophrenia 3 Anorexia nervosa 3	Pharmacological treatment 26	CGI-S Y-BOCS	April–May 2020	OCD patients have been only minimally impacted by COVID-19 restrictions. 3% experienced the deterioration of the OC symptom severity
Pan et al. (60), Netherlands	Analyse the perceived mental health impact, others variables and worry before and during the COVID-19 pandemic between people with and without lifetime depressive, anxiety, or OCD	Longitudinal study	*N* = 1,517 (285 with OCD), Age: 18–93 (13.2)	36	*NS*	Mental health treatment 605	PSWQ	April–May 2020	OCD patients show a slight symptom decrease

* *Wide Age Range; NS, Not Specified; OC, Obsessive-Compulsive; OCD, Obsessive-Compulsive Disorder; COVID-19, Coronavirus Disease 2019; NIMH-GOCS, National Institute of Mental Health-Global Obsessive-Compulsive Scale; OCI-R, Obsessive–Compulsive Inventory-Revised; Y-BOCS-SC Yale–Brown Obsessive Compulsive Symptom Scale Symptom Checklist; Y-BOCS, Yale-Brown Obsessive-Compulsive Scale; CY-BOCS, Children's Yale-Brown Obsessive-Compulsive Scale; CGI-S, Clinical Global Impression Severity; CGI-I, Clinical Global Impression–improvement; CBT, Cognitive behavioral therapy; ACT, Acceptance and Commitment Therapy; OCI-CV, Obsessive-Compulsive Inventory-Child version questionnaires; VOCI, Vancouver Obsessional-Compulsive Inventory; OCS, Obsession with COVID-19 Scale; PSWQ-A, Penn State Worry Questionnaire-Abbreviated; DOCS, Dimensional Obsessive–Compulsive Scale; PSWQ, The 11-item Penn State Worry Questionnaire; ERP, Exposure and Response Prevention*.

**Table 2:** Studies among sample of children and adolescents.

**References/Country**	**Aims/Purpose**	**Research design**	**Sample characteristics**	**% males**	**Comorbidity**	**Treatment**	**Outcome/measures**	**Monitoring period**	**Main findings**
Tanir et al. (48), Turkey	Investigate the effects of COVID-19 pandemic on symptom profile, symptom severity and exacerbation of OCD symptoms and related factors	Cross sectional study	*N* = 61, Age: 6–18	55.7	*NS*	Pharmacological treatment 47 CBT and SSRI 6 CBT 1	CY-BOCS CGI-S Phone interview	March–April 2020	Increase in the frequency of contamination obsessions and cleaning/washing compulsions during pandemic period
Nissen et al. (47), Denmark	Examine how children/adolescents with OCD react toward COVID-19 crisis	Cross sectional study	*N* = 65 clinical group newly diagnosed with OCD *N* = 37 survey group primary OCD treatment completed Age: 7–21 (14.9)	36.9 33.3	Clinical group: Others psychiatric disorder 42 Survey group: Others psychiatric disorder 19	Clinical group: Psychological therapy 41 SSRI 29 Neuroleptic 10 ADHD medication 7 Survey group: Psychological therapy 25 Pharmacological treatment 12	Qualitative questionnaire	April–May 2020	Worsening of their OCD, anxiety, and depressive symptoms: most in the survey group primary
Schwartz-Lifshitz et al. (61), Israel	Evaluate whether OCD exacerbated during the first wave of COVID-19 in children and adolescents	Cross sectional study	*N* = 29, Age: 14–19 (14.2)	65	Anxiety disorder 12	Psychological therapy 12 SSRI 6	CGI-S CGI-I OCI-CV	April–May 2020	OC symptoms were not found to have exacerbated during the period investigated

*NS, Not Specified; OC, Obsessive-Compulsive; OCD, Obsessive-Compulsive Disorder; COVID-19, Coronavirus Disease 2019; NIMH-GOCS, National Institute of Mental Health-Global Obsessive-Compulsive Scale; OCI-R, Obsessive–Compulsive Inventory-Revised; Y-BOCS-SC Yale–Brown Obsessive Compulsive Symptom Scale Symptom Checklist; Y-BOCS, Yale-Brown Obsessive-Compulsive Scale; CY-BOCS, Children's Yale-Brown Obsessive-Compulsive Scale; CGI-S, Clinical Global Impression Severity; CGI-I, Clinical Global Impression–improvement; CBT, Cognitive behavioral therapy; ACT, Acceptance and Commitment Therapy; OCI-CV, Obsessive-Compulsive Inventory-Child version questionnaires; VOCI, Vancouver Obsessional-Compulsive Inventory; OCS, Obsession with COVID-19 Scale; PSWQ-A, Penn State Worry Questionnaire-Abbreviated; DOCS, Dimensional Obsessive–Compulsive Scale; PSWQ, The 11-item Penn State Worry Questionnaire; ERP, Exposure and Response Prevention*.

### Assessment of Methodological Quality

To evaluate the quality of the studies was used a modified version of the Newcastle-Ottawa scale [NOS; (62–65)] adjusted for cross sectional and longitudinal studies. This instrument has a practical checklist that estimates the global quality as well as particular characteristics of the specific studies.

**Figure 2:**
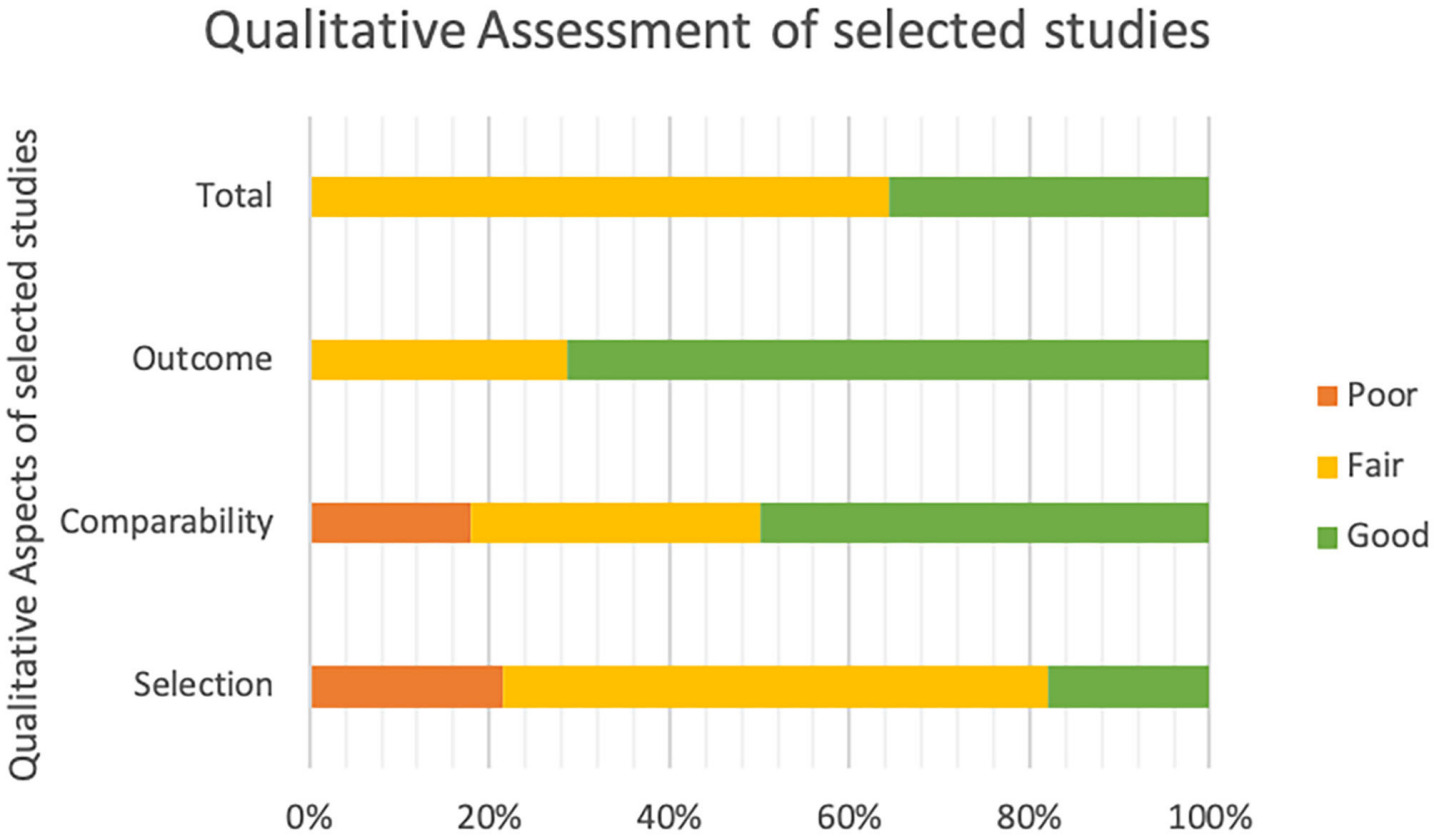
Qualitative assessment of the 14 studies included in the narrative review. Selection: representativeness and sample size; Comparability: correspondence of the variables between age and gender; Outcome: consistency of instruments used and relevance of statistical analyses; Total: Total Quality Score.

In particular, aspects such as selection (e.g., representativeness and sample size), comparability (i.e., correspondence of the variables between age and gender), and outcome (i.e., consistency of instruments used and relevance of statistical analyses) can be rated as good, fair, or poor. Three authors TC, AM, and GA made autonomous quality ratings, and disagreements were solved through discussion and consultation with other authors (VZ and MD'A). Figure 2 summarizes the quality of the studies included in the narrative review.

## Results

### Impact of COVID-19 on OCD in Children, Adolescents and Adults

The literature referring to the impact of COVID-19 on OCD is scarce. There are several studies relating to the impact on the adult population but less attention has been paid to children and adolescents specifically (47, 48, 61). From examination of the 14 studies reported (Tables 1 and 2), 10 documented a negative impact of COVID-19 on OCD (8 in adults and 2 in children and adolescents).

In detail we can observe, in adults, a clinical worsening in OC symptoms (51, 56, 58), an increase in contagion obsessions and washing compulsions (52, 53, 55), an increase in the symptoms of washing compulsions and avoidance behaviours p < 0.001) (52), a greater demand for psychiatric emergency (p = 0.003) for OCD patients with substances abuse and higher psychiatric emergency consultation during the lockdown in OCD patients compared to the previous year (53). Two studies (44, 59) found a minimal exacerbation of OC symptomatology.

Changes in the general severity of obsessions and compulsions (*p* < 0.001) are found by comparing the periods before and after the pandemic (53), finding that new phenotypes and the exacerbation of existing obsessions (*p* < 0.005) and compulsions (*p* < 0.001) (56) emerged. However, an important limitation of this study is that the new phenotypes are unknown. Furthermore, 1 study on adults (60) showed slight symptom improvement (*p* < 0.0001).

Moreover, we can observe in children and adolescents that the presence of poor insight and obsessions with aggressive content predict a worsening outcome (*p* = 0.02) (47). Furthermore, a significant increase in the frequency of contamination obsessions (*p* = 0.008) and cleaning and washing compulsions (*p* = 0.039) during the pandemic was found in a study involving children and adolescents (6–18 years), including those in psychological treatment or cognitive behavioral therapy (48). This is supported by Nissen et al. (47), who found an exacerbation of OCD in children and adolescents aged 7–21 years in treatment. The aggravation of OCD correlated with the worsening of anxiety, depressive symptoms and the extent of avoidance behavior. Moreover, OCD aggressive symptoms and poor baseline insight predicted a significant worsening. On the other hand, Schwartz-Lifshitz et al. (61) did not detect any exacerbation of OC symptoms during the first wave of COVID-19 in a sample aged 14–19 years and about half of the sample received no treatment.

### Typology of OC Symptoms

Much of the literature has focused on overall symptoms of OCD, without analyzing the differences between subtypes (contamination/washing, checking, symmetry and forbidden thoughts). Of the 14 studies examined, 10 of these addressed the issue of OCD subtype (7 in adult patients and 3 in children and adolescents); however, 4 studies did not investigate the relation between specific OCD domains and COVID-19 (51, 54, 59, 60).

In a study on German adult patients, the authors split the initial sample into participants with and without washing compulsions and found an increase in the severity of OCD particularly for patients of the washing subtype (52). Similarly, Prestia et al. (53) found that patients with contamination symptoms had a significantly stronger worsening of the severity of OCD (time spent, degree of interference, distress, resistance, and perceived control over symptoms) from before quarantine to the quarantine period.

Again, Tanir et al. (48) examined symptom severity before and during the COVID-19 pandemic in a sample of children and adolescents with OCD; they showed a significant increase in the frequency of contamination obsessions and washing compulsions.

In agreement with these results Khosravani et al. (57) observed, in a sample of Iranian OCD patients, higher COVID-19-related stress responses, such as fear of danger and contamination, socio-economic consequences, xenophobia, traumatic stress, and compulsive behaviors of checking and reassurance-seeking. Matsunaga et al. (55) showed that 10% of patients in full or partial remission experienced deterioration in the symptom severity of OCD, and almost all these subjects had primary OCD symptoms associated with contamination/washing; just one subject had symptoms of symmetry/repeating and ordering type. Furthermore, a small portion of the sample with aggressive/checking and symmetry/repeating and ordering OCD showed additional symptoms such as contamination obsessions or washing compulsions, but no subjects showed symptom transition of their core symptoms. Kuckertz et al. (58) reported eight cases of patients with different core symptoms: symmetry, washing, harm obsessions or intrusive thoughts. One of these patients (with concerns around perfectionism, intrusive thoughts and contamination) reported COVID-19-related stressors and increases in anxiety throughout the pandemic; however, the impact in terms of increased specific symptoms remains unclear. Likewise, a sample of Italian adult patients experienced an increase in avoidance behaviors mostly related to the fear of possible contamination, but information about specific symptom domains is not provided (56).

Conversely, in a study on children and adolescents, Nissen et al. (47) found no link between COVID-19 and washing compulsion but discovered that the occurrence of baseline aggressive/sexual thoughts and rituals increased the risk of experiencing a worsening of OCD symptoms. However, Schwartz-Lifshitz et al. (61), in a sample of adolescents, and Chakraborty and Karmakar (44), in a sample of patients of unspecified age, did not find any exacerbation of OCD during the COVID-19 pandemic.

### Studies Characteristics

Studies showed 3 different researcher's design: 8 cross sectional studies, 5 longitudinal studies, 1 preliminary naturalistic study (Tables 1, 2).

Outcomes were collected through different methodology such as quantitative measures (self-report, questionnaire, semi-structured interview), online survey [e.g., (51, 52)], phone and in person interview [e.g., (53, 54)] or video call [e.g., (44)]. In a single study data were provided from clinician's opinion [e.g., (51)].

Studies were conducted during the first lockdown period corresponding from January to May 2020 in all the countries. Just 2 studies (51, 57) reported outcomes obtained in the monitoring period June –August 2020.

Furthermore, 1 study [e.g., (54)] compared outcomes from 2019 to other data picked up during first lockdown (January–May 2020).

### Sample Characteristics

In general, the study samples had heterogeneous characteristics such as gender, age and comorbidity. Studies included both small samples [*N* = 8: (58); *N* = 29: (61)] and larger-scale trials [*N* = 394: (52); *N* = 300: (57)]. In other studies, there was a large sample but a comparatively small range of people with OCD [*N* = 1,517 in total and *N* = 285 with OCD: (60)] or a small sample but a comparatively large percentage of people with OCD [*N* = 30 in total and *N* = 12 with OCD: (59)].

In a cross sectional study by Nissen et al. (47) there were two samples: a clinical group newly diagnosed with OCD (*N* = 65) and a survey group with primary OCD treatment completed (*N* = 37). In the study by Matsunaga et al. (55) there were also two samples: fully remitted and partially remitted patients. Moreover, in a longitudinal study by Jelinek et al. (52) there were two specific OCD subsamples, washers (*N* = 223) and not-washers (*N* = 171), in order to compare the differences between people with compulsions during the COVID-19 pandemic.

Study participants were OCD subjects of both genders with different comorbidities, such as anxiety disorder (51, 61), depressive disorder (51), mood disorder and personality disorders (53). In other studies, psychiatric comorbidity was not specified (56) or not present [e.g., (55)].

The age in some studies was not declared (44, 58). Only 3 studies included OCD samples among children and adolescents: ages 6–18 years (48), 14–19 years (61), and 7–21 years (47). Other studies included samples with a wide age range, particularly the longitudinal study conducted by Storch et al. (51) (ages 4–77 years). Among the 14 studies analyzed, 13 did not specify the ethnicity of the patients, whereas in an Italian cross sectional study, authors reported that 13.9% of first sample and the 12.9% of second sample, was not Italian (54).

Regarding studies samples, in only 1 study it is possible to observe patients with OC symptoms also affected by COVID-19 (60).

### Measurements

Many of the selected studies used similar or homogeneous quantitative measures in order to reveal any subjective or effective exacerbation of OC symptoms. These instruments were identified and chosen by the research community for their excellent psychometric properties. However, not all the analyzed studies used measures with demonstrated treatment sensitivity and good reliability. Actually, some articles [e.g., in Benatti et al. (56)] opted for non-specific psychometric assessment and used qualitative instruments such as surveys (52) or non-validated questionnaires (47).

Concerning adult samples, almost 60% of the selected articles used the same tool administered by clinicians: the Yale-Brown Obsessive Compulsive Scale [Y-BOCS; (66)]. The Y-BOCS is a ten-item measure considered to be the gold standard for OCD symptom severity. It is a reliable semi-structured interview, split into subscales for obsessions and compulsions. The five categories of obsessive and compulsive symptoms are rated on a scale from 0 (no symptoms) to 4 (extreme symptoms): time spent, degree of interference, distress, resistance (greater resistance is assigned lower scores), and perceived control over symptoms. Subscale scores are added to obtain the total scores. In the present narrative review it has been used generally in its integral version [e.g., in Chakraborty and Karmakar (44)], in its children's form [CY-BOCS; (47, 48, 67)] or by adapting a few of its questions (51).

In addition, a new measure—the COVID Stress Scales (68)—was designed to assess contamination fears and compulsive checking due to COVID-19-related danger (57).

Different studies among adult samples opted for self-report measures such as the Obsessive-Compulsive Inventory-Revised [OCI-R; (69)], used by Jelinek et al. (52) and Khosravani et al. (57).

Two studies (54, 56) used only qualitative instruments to assess OCD worsening, such as a general psychiatric interview and questions to identify the main phenotypes of obsessions and compulsions (56). Other researchers support quantitative with qualitative data, adopting *ad hoc* questionnaires to identify the severity of OCD, changes in symptoms since the beginning of the COVID-19 pandemic (52) and quality of life during quarantine (53).

For studies focused on young subjects, the Children's Yale-Brown Obsessive Compulsive Scale [CY-BOCS; (67)] was used, which is a semi-structured clinician-rated instrument similar to the adult version [Y-BOCS; (66)], but generally different tools were adopted. In detail, 2 studies (48, 61) employed the Clinical Global Impression scale and it was used in its Improvement and Severity Subscales [CGI-I and CGI-S; (70)]. CGI is a measure used to assess the symptom profile and rate OCD severity. Schwartz-Lifshitz et al. (61) included in their research a validated self-report questionnaire, the Obsessive-Compulsive Inventory-Child Version [OCI-CV; (71)], which provides seven scores: Doubting-Checking, Obsessing, Hoarding, Washing, Ordering and Neutralizing.

With regard to how the instruments were used, in order to be in line with government and health service policy (https://www.gov.ie/en/speech/f27026-speech-of-an-taoiseach-leo-varadkar-td-government-buildings-27-march/), half of the analyzed articles opted for online methods, such as phone interviews, online surveys and, whenever possible, video calls (44). Most of the interviews were conducted by telephone or online because of the additional stress associated with an in-person interview for OCD patients who could have contamination fears.

### Types of Treatment

In the studies analyzed, the samples received different types of treatment: pharmacological treatment, cognitive behavioral therapy (CBT), acceptance and commitment therapy (ACT), exposure and response prevention (ERP) and psychological support. Only 2 studies involved patients who received ERP treatment during the pandemic period (51, 58). In 9 studies, some of the sample was in pharmacological treatment. For example, in the study by Chakraborty and Karmakar (44) 57 subjects took medicines regularly, 13 subjects took them intermittently and 4 subjects had stopped taking their medicines. In another study (56), 123 subjects were in pharmacological treatment; and in Plunkett et al. (59), from a total sample of 30 individuals there were 26 subjects in pharmacological treatment.

A study conducted by Prestia et al. (53) shows that all patients were on stable pharmacological treatment during the last 6 months before quarantine and some of them also had CBT. Other samples received pharmacological treatment that was not specified (58) and for others the presence of treatment was not reported (52, 54, 55). Furthermore, children and adolescent samples had different types of treatment: pharmacological treatment; psychological therapy that was not specified; CBT; and CBT and medical treatment.

In the study by Tanir et al. (48), 47 subjects received only pharmacological treatment, 6 subjects received CBT and a selective serotonin reuptake inhibitor (SSRI), 1 subject received only CBT and 7 received no treatment. In another study (47) 41 subjects of the clinical group were in a psychological therapy that was not specified; regarding pharmacological treatment, 29 were taking SSRI medication, 10 were on neuroleptics and 7 were on ADHD medication. Only 25 subjects of the survey group received therapy at the time of the questionnaire and 12 were treated with SSRI medication.

In the study by Schwartz-Lifshitz (61), all subjects were treated with psychiatric and/or psychotherapeutic treatment. Twelve participants (42%) received psychotherapeutic intervention during the study period and the majority of participants (19; 65%) were treated with an SSRI.

## Discussion

Our paper aimed to analyze and review all the empirical contributions investigating the impact of the COVID-19 pandemic on OC symptoms in children, adolescents and adults with OCD. The coronavirus pandemic has had an impact on OCD in both adults and young people: COVID-19, in fact, seems to have caused an exacerbation of symptoms, especially of the contamination/washing subtype (49).

It is plausible to speculate that constant warnings about coronavirus and incessant reminders to keep high levels of hygiene may have exacerbated obsessive fears related to contamination (72).

Nevertheless, only 14 studies have gone through the reviewing process and some of these report controversial results.

Interestingly, despite the medications and the possibility of being in psychological treatment, adult participants of eight studies showed an increase in the severity of OC symptoms. However, 3 different studies underlined only a minimal impact of COVID-19 on OCD patients and in one study the patients even showed slight symptom improvement (60). In detail, Kuckertz et al. (58) also underlined that, for some patients, COVID-19 was an interesting opportunity to be more fully engaged in exposure. On the other hand, 2 studies on children and adolescents show a worsening even in the presence of ongoing treatment (47, 48) and in one study there is no exacerbation of symptomatology, probably due to psychological and pharmacological therapy. However, worsening of OC symptoms, as seen in the other 2 studies, seems to be the most frequent result even though young participants were on CBT or in pharmacological treatment.

It is important to underline such as current treatment could influence the results regarding the change of OC symptomatology because in most studies the typology of treatment is not clear or only some patients are treated.

In effect, data on the type and frequency of treatment are unclear and heterogeneous. Most of the studies analyzed did not offer a clear picture of the type of treatment utilized in all the samples. There was a prevalence of different pharmacological treatments and psychological therapies but without any explicit specification of the program.

Moreover, only few studies examined the problem of OCD subtypes. In most cases, both in adults and in adolescents and children, these studies have shown an exacerbation of the symptoms of contamination/washing subtype and in one case an effect on aggressive/sexual thoughts. However, there are also conflicting results that show no effect of the pandemic on specific OCD domains.

Regarding the measures used in data collection, all the selected articles opted for homogeneous quantitative measurements with excellent psychometric properties and/or qualitative instruments, such as surveys or non-validated questionnaires. Among the adult samples, the Y-BOCS (66) has often been used and it is important to consider that it is a valid measurement, gold standard for the severity of OCD symptoms. Its children's form, the CY-BOCS (66), was adopted among young participants, as well as the CGI-S (70). Concerning self-report measures, the OCI-R (69) and the OCI-CV (71) were, respectively, used in adult and young samples. Half of the analyzed articles opted for online methods, such as phone interviews and online surveys, in order to be in line with government and health policy.

However, it is essential to consider that this qualitative data collection procedure or use of non-standardized quantitative measures could be a limitation in the convergence of data. Although, as reported in studies characteristics, measurements heterogeneity could probably affect results, for example, an in-person interview directed by clinicians could be more reliable than a phone interview or a self-report questionnaire.

Moreover, regarding the characteristics of the samples, it is detected a wide age range that did not allow the different effects among age groups to be fully differentiated.

All studies have been conducted during some monitoring period, except 2 studies carried out during the summer after first lockdown. We assume that this difference between monitoring period could not excessively influence outcomes. Instead, we consider an absence of results for long period more influent to really understand COVID-19 impact on OC symptoms.

Furthermore, it is important to point out that in the different studies it is not documented whether the participants had COVID-19 during the pandemic period. The absence of this data, given the historical period and the distress experienced, does not allow to evaluate a possible specific weight of this variable on the mental and clinical state of the participants with OCD. It can be expected that OCD patients exposed to friends/family affected by COVID-19 (48, 53) or to medical staff infected (58) could have a worsening experience of their symptoms.

With such study samples, it is impossible to understand if there was a different impact on OCD symptoms in children and adults during the COVID-19 pandemic owing to the heterogenous characteristics.

It is essential to highlight how the different characteristics of the samples, the heterogeneity in the information collection procedure, the recruitment process, the different phases in the representation of symptoms during the pandemic are essential aspects that influence the reliability of the results.

It seems essential to consider these aspects as they are controversial variables that make it difficult to compare or to rely on the results.

Finally, these results indicate the need to enrich the literature in this area considering the bias present, with particular attention to OCD children and adolescents as the contributions are scarce compared to the adult clinical population.

## Conclusion

The number of studies reviewed is quite small, there are more studies on adult OCD samples than on populations of children and adolescents and emerge some controversial results: few studies examined OCD subtypes; in most studies the typology of treatment was not clear and the samples covered a wide age range; a number of studies did not use the same monitoring period or quantitative measures, both of which make it difficult to compare or rely on the results.

In conclusion, the findings are hard to interpret due to the numerous types of treatments and measurements and the heterogeneity of the samples.

The most information was registered from Italy and United States. Italy had built up the major number of results and contributes on this topic.

Our results indicate the need to enrich this field of study and appears important for clinicians and the scientific community to shed light on the impact of this event on OCD, a psychiatric disorder that causes significant impairment in general functioning. This knowledge is fundamental to make use of more appropriate and timely interventions in clinical practice and understand how contextual variables can exacerbate some OC symptoms both in adult population and in development age.

## Limitations

This narrative review has some limitations: no systematic review process has been carried out; it was performed only in the clinical population and work on OC symptoms with other comorbidities or in the general population was not included; and other variables present in the studies (such as worsening with other clinical profiles, etc.) were not commented on or summarized. Notwithstanding these limitations, the collective findings in the current study highlight the need to conduct studies to address the research gaps and to better understand the impact of COVID-19 in the OCD population in order to ensure the availability of studies in the literature.

## Author Contributions

VZ took overall responsibility for the conceptualization and design of the review and revised it critically for important intellectual content. VZ, MD'A, TC, AM, and GA, searched for the articles in the review, assessed them for relevance, interpretation of data, in writing and editing the final article, final approval of the version to be published, and agreement to be accountable for all aspects of the work in ensuring that questions related to the accuracy or integrity of any part of the work are appropriately investigated and resolved. All authors contributed to the article and approved the submitted version.

## Conflict of Interest

The authors declare that the research was conducted in the absence of any commercial or financial relationships that could be construed as a potential conflict of interest.
